# Hydrogen Peroxide Mediates Premature Senescence Caused by Darkness and Inorganic Nitrogen Starvation in *Physcomitrium patens*

**DOI:** 10.3390/plants11172280

**Published:** 2022-08-31

**Authors:** Md. Shyduzzaman Roni, Md. Arif Sakil, Most Mohoshena Aktar, Chihiro Takatsuka, Kyosuke Mukae, Yuko Inoue-Aono, Yuji Moriyasu

**Affiliations:** 1 Graduate School of Science and Engineering, Saitama University, Saitama 338-8570, Japan; 2 Department of Horticulture, Bangabandhu Sheikh Mujibur Rahman Agricultural University, Gazipur 1706, Bangladesh; 3 Department of Biochemistry and Molecular Biology, Bangladesh Agricultural University, Mymensingh 2202, Bangladesh; 4 Department of Agronomy, Hajee Mohammad Danesh Science and Technology University, Dinajpur 5200, Bangladesh; 5 School of Humanities, Tokai University, Shizuoka 424-8610, Japan; 6 Research Institute for Clinical Oncology, Saitama Cancer Center, Saitama 362-0806, Japan

**Keywords:** senescence, methyl viologen, nitrogen starvation, dark, H_2_O_2_, *Physcomitrium*

## Abstract

Leaf senescence accompanied by yellowing and Rubisco degradation occurs prematurely in response to various stresses. However, signaling pathways between stress perception and senescence responses are not understood fully, although previous studies suggest the involvement of reactive oxygen species (ROS). While investigating the physiological functions of autophagy in *Physcomitrium patens* using wild-type (WT) and autophagy-deficient *atg5* strains, we found that *Physcomitrium* colonies senesce prematurely under dark or nitrogen-deficient conditions, with *atg5* senescing earlier than WT. In the present study, we measured cellular H_2_O_2_, and examined whether H_2_O_2_ mediates premature senescence in *Physcomitrium* colonies. Methyl viologen, an ROS generator, increased cellular H_2_O_2_ levels and caused senescence-like symptoms. H_2_O_2_ levels were also elevated to the same plateau levels in WT and *atg5* under dark or nitrogen-deficient conditions. The ROS scavenger N-acetylcysteine and the ROS source inhibitor carbonyl cyanide m-chlorophenylhydrazone inhibited the increase in H_2_O_2_ levels as well as senescence. Upon transfer to a nitrogen-deficient medium, H_2_O_2_ levels increased earlier in *atg5* than in WT by ~18 h, whereas *atg5* yellowed earlier by >2 days. We conclude that the increased H_2_O_2_ levels under dark or nitrogen-deficient conditions mediate premature senescence in *Physcomitrium* but do not explain the different senescence responses of WT and *atg5* cells.

## 1. Introduction

Leaf senescence is a degenerative process in which leaf cells and tissues are degraded. During senescence, cellular components, including chlorophyll and the Rubisco protein, are degraded, and the degradation products, including amino acids and sugars, are transported from the senescing leaf to adjacent or distant organs. Leaves typically senesce after reaching maturity; however, leaf senescence can be induced prematurely by a variety of stresses, such as darkness, nutrient starvation, drought, and salinity.

Autophagy is one of the pathways in which cells degrade their own components. During autophagy, a portion of the cytoplasm is transported to lysosomes and/or vacuoles, where it is degraded [1]. The various autophagy types include macroautophagy, microautophagy, and chaperon-mediated autophagy, of which macroautophagy is widely characterized. In macroautophagy (hereafter referred to as autophagy), cytoplasmic components are enclosed in an autophagosome, which fuses with a lysosome or vacuole and is degraded into amino acids and nucleotides. The amino acids are reused in protein synthesis or energy production through oxidation. Autophagy is executed and controlled by various autophagy-related (Atg) proteins [2].

The signaling pathways in premature senescence from stress perception to senescence responses are not fully elucidated. However, previous studies have suggested that reactive oxygen species (ROS) are involved in these pathways. First, it has been reported that increased ROS levels mediate the premature senescence of leaves in plants under drought stress [3]. Specifically, the cellular H_2_O_2_ level increases and premature senescence occurs in *Arabidopsis* leaves under drought stress; however, in *Arabidopsis* mutants deficient in the expression of ROS-generating enzymes, H_2_O_2_ levels are reduced and senescence is delayed [3]. In addition, increased H_2_O_2_ levels increase during NaCl-induced senescence in sweet potato have been reported [4]. The study also found that a calmodulin inhibitor decreased H_2_O_2_ levels and inhibited premature senescence [4]. Second, cellular H_2_O_2_ levels are shown to increase during natural senescence in *Arabidopsis* and oilseed rape, and senescence is delayed by reducing H_2_O_2_ levels through the overexpression of exogenous proteins that scavenge H_2_O_2_ [5,6]. Third, increased H_2_O_2_ levels are observed in *Arabidopsis* autophagy-deficient mutants that exhibit earlier senescence [7,8]. In addition, several studies have indicated that cellular ROS levels are elevated in response to abiotic and biotic stress [9,10]. Collectively, these results suggest that ROS, especially H_2_O_2_, are mediators between stress perception and senescence responses.

We have been investigating the physiological significance of autophagy in plants using the moss *Physcomitrium patens*. This moss has the property of undergoing homologous recombination with DNA introduced into cells with high efficiency, and techniques for disrupting specific genes have been developed [11]. We also reasoned that the simple cellular structure and few cell types of *Physcomitrium* protonema colonies would facilitate physiological analysis at the cellular level. In a previous study, we constructed *ATG5* gene knockout (*atg5*) mutants and found that *Physcomitrium* protonema colonies show stress-induced senescence symptoms similar to those in green leaves and that *atg5* mutants senesce earlier than the wild-type (WT) under dark or nitrogen starvation conditions [12].

In the present study, we investigated whether H_2_O_2_ is a mediator between the perception of stress, i.e., darkness and inorganic nitrogen starvation, and senescence responses, including yellowing and Rubisco degradation, in WT and *atg5* mutants of *Physcomitrium*. As we found that H_2_O_2_ acts as a mediator of premature senescence, we further investigated whether increased H_2_O_2_ levels account for the difference in senescence responses between WT and *atg5* cells. However, we found that increased H_2_O_2_ did not explain the earlier premature senescence phenotype of *atg5* mutants that occurred under dark or nitrogen starvation conditions.

## 2. Results

### 2.1. Premature Senescence Is Induced by Either Darkness or Inorganic Nitrogen Starvation in Physcomitrium

**Figure 1:**
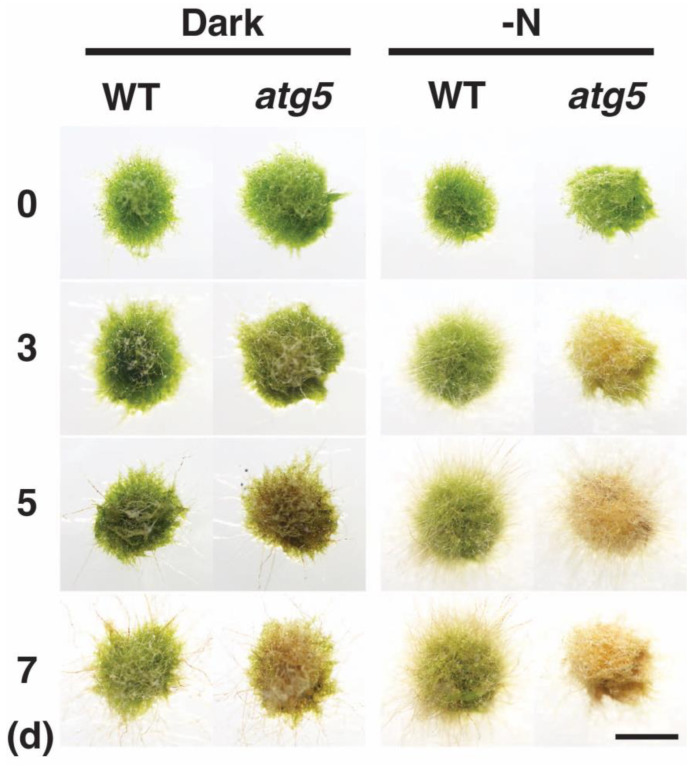
Senescence of wild-type (WT) and *atg5* mutant *Physcomitrium* colonies under dark and nitrogen starvation conditions. WT and *atg5* colonies were transferred onto and cultured on a nutrient-sufficient BCDATG agar medium in the dark (**Dark**) or on inorganic nitrogen-depleted BCDATG agar medium under light conditions (**-N**) for 7 d. Individual colonies in each treatment group were photographed immediately (0 d) and 3, 5, and 7 d after transfer. In–N, the same colonies were photographed successively for 7 d. Scale bar: 2 mm.

*Physcomitrium* colonies consisting of protonemal cells senesce prematurely when they are placed under dark conditions, and autophagy-deficient *atg5* mutants senesce earlier than the WT strain [12]. The transfer of these colonies to a medium lacking an inorganic nitrogen source also induces premature senescence, which again occurs earlier in the *atg5* strain than in the WT strain. Here we compared darkness- and nitrogen-starvation-induced premature senescence in these strains (Figure 1). When kept in the dark, WT colonies became yellowish on day 7, whereas *atg5* colonies became yellowish on day 3–5 and brown on day 7 (Figure 1, **Dark**). When kept on inorganic nitrogen-deprived agar medium under light conditions, WT colonies became yellowish on day 5–7, whereas *atg5* colonies became yellowish on day 3 and brown on day 5–7 (Figure 1, **-N**). Thus, nitrogen-starvation-induced senescence proceeded earlier than dark-induced senescence, and the *atg5* mutant senesced earlier than the WT strain in each senescence process.

### 2.2. Methyl Viologen Induces Senescence-Like Symptoms

**Figure 2:**
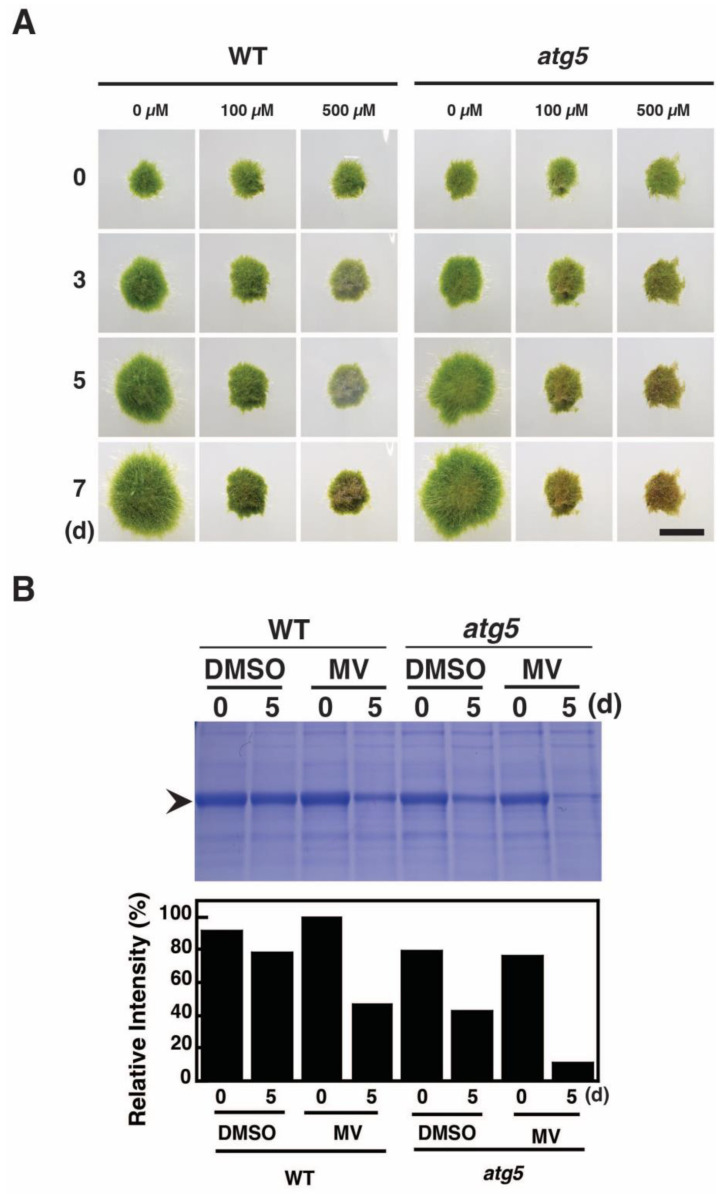
Methyl viologen (MV) treatment causes senescence-like symptoms in WT and *atg5* mutant *Physcomitrium* colonies cultured on a nutrient-sufficient agar medium. (**A**) WT and *atg5* colonies were transferred onto and cultured on a BCDATG agar medium containing methyl viologen (MV; 0, 100, or 500 µM) for 7 d under light conditions. Individual colonies in each treatment group were successively photographed immediately (0 d) and 3, 5, and 7 d after transfer. Scale bar: 2 mm. (**B**) (Top) Water-soluble proteins were extracted from WT and *atg5* colonies immediately (0 d) and 5 d after the transfer onto BCDATG agar medium containing MV (100 µM) or DMSO (as a solvent control) and analyzed using SDS-PAGE. The gel was stained with Coomassie brilliant blue. Proteins were loaded into each lane based on fresh weight. Arrowhead, Rubisco large subunit. (Bottom) Rubisco large subunit levels were estimated using densitometry and Image J (imagej.nih.gov/ij/download/).

Methyl viologen (MV) is thought to produce toxic effects in plants by generating ROS that are converted into H_2_O_2_ in cells [13]. To examine the effects of H_2_O_2_ on *Physcomitrium* colonies, 7-d-old WT and *atg5* colonies were placed on nutrient-sufficient agar medium containing MV and cultured under light conditions. MV at 100 μM inhibited the growth of *atg5* colonies and caused yellowing on day 5 (Figure 2A), which progressed until day 7. WT colonies also stopped growing on the medium containing MV at 100 μM, but they remained green until day 7 with only a small yellow portion observed on this day. When the MV concentration was increased to 500 μM, the yellowing occurred more rapidly in *atg5* colonies, and the WT colonies showed yellowing on day 3 that proceeded until day 7. Thus, MV causes yellowing in *Physcomitrium* colonies, and this effect is more pronounced in *atg5* colonies compared with that in WT colonies at the same MV concentration.

Additional experiments revealed that *atg3* and *atg7* mutants showed a similar MV-induced yellowing response to that of *atg5* mutants (Supplemental Figure S1). In contrast, *ATG5* mutants, in which the *Phycomitrium ATG5* gene was introduced into the *atg5*-3 mutant, exhibited a similar response to that observed in the WT strain (Supplemental Figure S1).

When the colonies were treated with MV in liquid medium under light, the yellowing response was the same as that exhibited on agar medium (Supplemental Figure S2, Light), whereas colonies placed in the dark exhibited dark-induced senescence (Supplemental Figure S2, Dark) and MV treatment had little effect on the progression of this senescence. This result is consistent with the notions that (i) the MV target site is the chloroplast electron transport chain (ETC) and (ii) light is required for the MV-induced production of ROS. Conversely, the result also supports the notion that yellowing is caused by MV-produced ROS under light conditions.

Premature senescence induced by darkness or nitrogen starvation is accompanied by a decrease in Rubisco content [12]. Thus, we also examined the effect of MV treatment on Rubisco content. WT and *atg5* colonies were transferred into liquid BCDATG medium containing MV and cultured under light conditions. Rubisco levels per fresh weight (FW) decreased significantly 5 d after MV treatment in both WT and *atg5* strains (Figure 2B, **MV**), although these levels were also reduced by the control treatment (**DMSO**) due to the increase in water content in the colonies upon initiation of liquid culture. Notably, the MV-induced decrement in Rubisco content was higher in *atg5* colonies than that in WT colonies, indicating that MV facilitates the net degradation of Rubisco and does so more drastically in the *atg5* mutant relative to the WT strain.

Taken together, these results show that MV treatment induces senescence symptoms, yellowing, and net Rubisco degradation in *Physcomitrium*.

### 2.3. MV Treatment Increases Cellular H_2_O_2_ Levels

**Figure 3:**
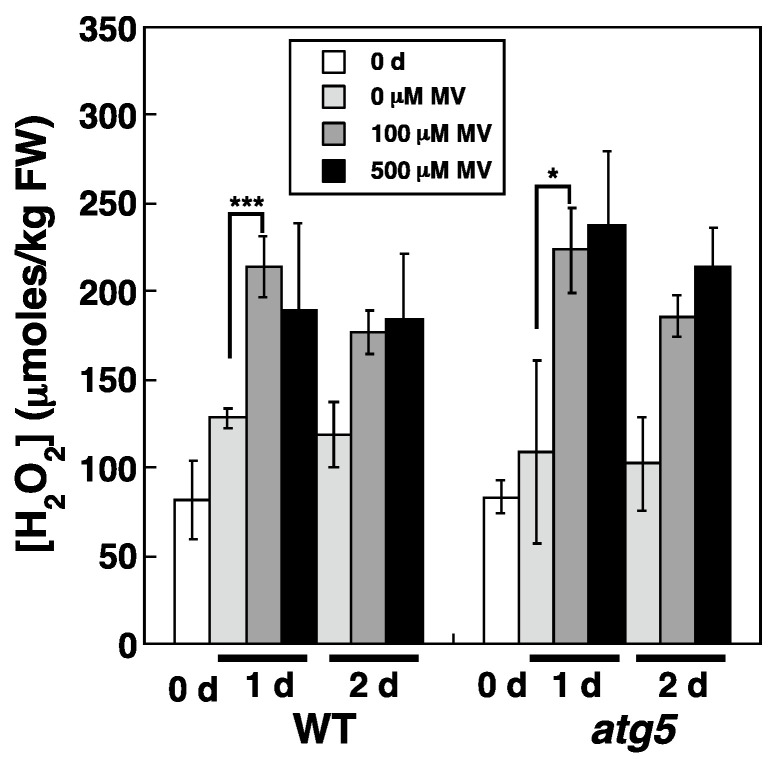
Effects of MV on changes in intracellular H_2_O_2_ levels in WT and *atg5* mutant *Physcomitrium* colonies cultured on a nutrient-sufficient agar medium. WT and *atg5* colonies were transferred onto and cultured on a BCDATG agar medium containing MV (0, 100, and 500 µM) under light conditions. Intracellular H_2_O_2_ levels were measured immediately (0 d) as well as 1 d, and 2 d after transfer. The data are represented as the means ± standard deviation (SD) (n = 3, *** *p* < 0.005, * *p* < 0.05).

To determine whether MV treatment increases cellular H_2_O_2_ levels, WT and *atg5* colonies were transferred onto BCDATG agar medium containing MV at 0–500 μM, and the subsequent changes in cellular H_2_O_2_ levels were measured. When WT and *atg5* colonies grown on nutrient-sufficient medium for 7 d were homogenized, the measured H_2_O_2_ level was 75–100 μmoles/kg FW, with no significant difference detected between the WT strain and *atg5* mutant (Figure 3, **0 d**). H_2_O_2_ levels tended to increase slightly in both strains after their transfer to fresh BCDATG agar medium lacking MV (Figure 3, **0 µM MV**); however, when MV was present in the medium, the H_2_O_2_ levels increased up to 200 μmoles/kg FW in both strains after 1 d and remained at similarly high levels after 2 d (Figure 3; **100 μM MV**, **500 μM MV**). Therefore, MV treatment increases cellular H_2_O_2_ levels in both the WT strain and *atg5* mutant. Notably, the H_2_O_2_ levels 1–2 d after MV treatment did not differ significantly between the two strains, although the yellowing phenotype in the *atg5* mutant was more pronounced than that in the WT strain.

### 2.4. Cellular H_2_O_2_ Levels Increase under Dark Conditions

**Figure 4:**
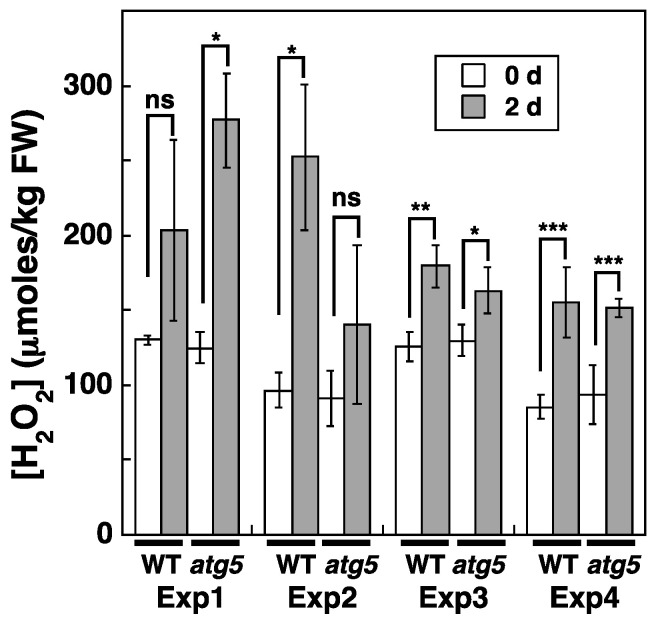
Changes in intracellular H_2_O_2_ levels in WT and *atg5 Physcomitrium* colonies cultured on a nutrient-sufficient medium in the dark. WT and *atg5* colonies were transferred onto and cultured on a fresh BCDATG agar medium under dark conditions. Intracellular H_2_O_2_ levels were measured immediately (0 d) and 2 d after transfer. The results were obtained from four independent experiments. The data are represented as the means ± SD (n = 3, * *p* < 0.05, ** *p* < 0.01, *** *p* < 0.005). ns, not significant.

We also investigated whether cellular H_2_O_2_ levels change during dark-induced senescence. To measure H_2_O_2_ concentrations, WT and *atg5* colonies were homogenized 2 d after being placed under dark conditions, i.e., 1 d before the appearance of the yellowing phenotype in the *atg5* mutant (Figure 4). Cellular H_2_O_2_ levels increased from 75–100 to ~150–250 μmoles/kg FW after 2 d under dark conditions. However, the H_2_O_2_ level reached after 2 d varied significantly among our experiments. This variability was thought to be caused mainly by the exposure of the colonies to laboratory light for 10–20 min to measure their FW before homogenization. Here, we show the results of four independent experiments (Figure 4), all of which indicate that cellular H_2_O_2_ levels increase under dark conditions in both WT and *atg5* colonies.

### 2.5. Cellular H_2_O_2_ Levels Increase Due to Inorganic Nitrogen Starvation

**Figure 5:**
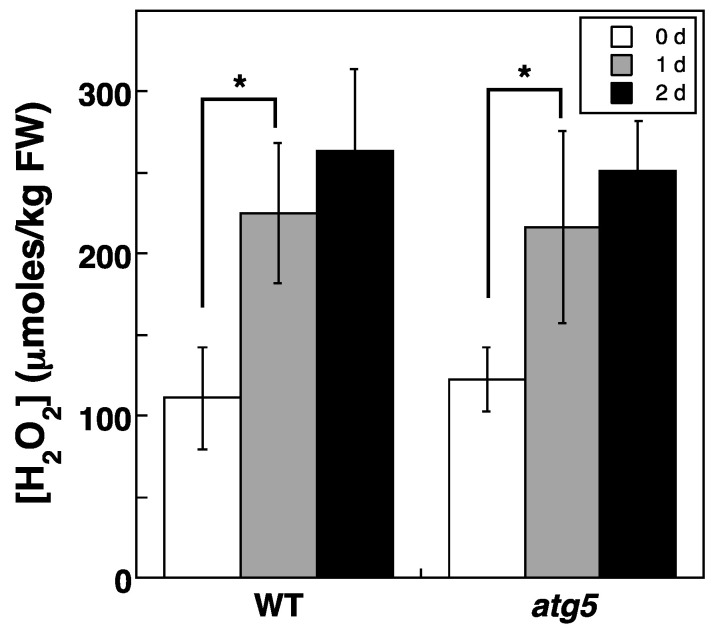
Changes in intracellular H_2_O_2_ levels in WT and *atg5 Physcomitrium* colonies cultured on an inorganic nitrogen-starvation medium. WT and *atg5* colonies were transferred onto and cultured on an inorganic nitrogen-depleted agar medium under light conditions. Intracellular H_2_O_2_ levels were measured immediately (0 d) as well as 1 d, and 2 d after the transfer. The data are represented as the means ± SD (n = 3, * *p* < 0.05).

Intracellular H_2_O_2_ levels also increased when colonies were placed on a nitrogen-starvation medium. Specifically, cellular H_2_O_2_ levels increased from around 100 to ~200 μmoles/kg FW after 1 d, and the increased levels lasted for at least 2 d (Figure 5). No significant difference in H_2_O_2_ levels was detected between WT and *atg5* colonies.

### 2.6. N-Acetylcysteine, an ROS Scavenger, Decelerates Yellowing

**Figure 6:**
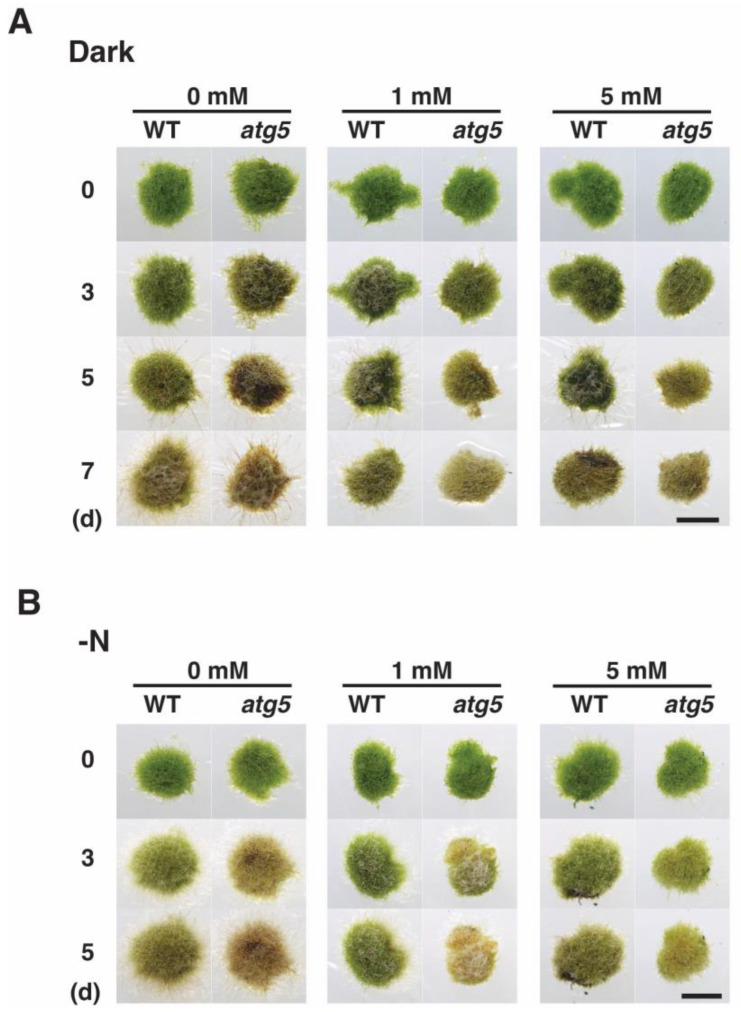
Effects of N-acetylcysteine (NAC) treatment on darkness- and nitrogen-starvation-induced senescence in WT and *atg5 Physcomitrium* colonies. (**A**) WT and *atg5* colonies were transferred onto and cultured on a BCDATG agar medium containing NAC (0, 1, and 5 mM) under dark conditions. Individual colonies in each treatment group were photographed immediately (0 d), 3, 5, and 7 d after the transfer. (**B**) WT and *atg5* colonies were transferred onto and cultured on an inorganic nitrogen starvation agar medium containing NAC (0, 1, or 5 mM) under light conditions. Individual colonies in each treatment group were successively photographed immediately (0 d), 3, 5, and 7 d after the transfer. Scale bars: 2 mm.

As our results suggested that darkness- and inorganic nitrogen deprivation-induced premature senescence is mediated by increased intracellular H_2_O_2_ levels, we investigated whether senescence is prevented or delayed by decreasing these levels. Specifically, we assessed the effects of the ROS scavenger N-acetylcysteine (NAC), which is thought to lower cellular ROS levels, on senescence. NAC treatment inhibited the progression of dark-induced senescence. In the absence of NAC, *atg5* colonies underwent yellowing after 3–5 d, whereas *atg5* colonies on a culture medium containing NAC at 1 mM or 5 mM remained visibly greener (Figure 6). WT colonies showed signs of yellowing on day 5 and browning on day 7 in the absence of NAC, but these effects were inhibited in WT colonies on culture medium containing NAC at 1 and 5 mM.

NAC also inhibited nitrogen-starvation-induced premature senescence. Treatment with NAC at 5 mM inhibited the yellowing of *atg5* colonies that typically manifested on days 3–5, whereas treatment with NAC at 1 mM only partially inhibited the yellowing of *atg5* colonies on days 3–5. The slight yellowing of WT colonies on days 3 and 5 was also inhibited by treatment with NAC at 1–5 mM. These results suggest that reducing H_2_O_2_ levels inhibits the progression of both darkness- and nitrogen-starvation-induced senescence, and they support the notion that increased ROS levels mediate the induction and/or progression of premature senescence in *Physcomitrium*.

### 2.7. Carbonyl Cyanide m-Chlorophenylhydrazone Lowers H_2_O_2_ Levels and Decelerates Senescence under Nitrogen Starvation Conditions

**Figure 7:**
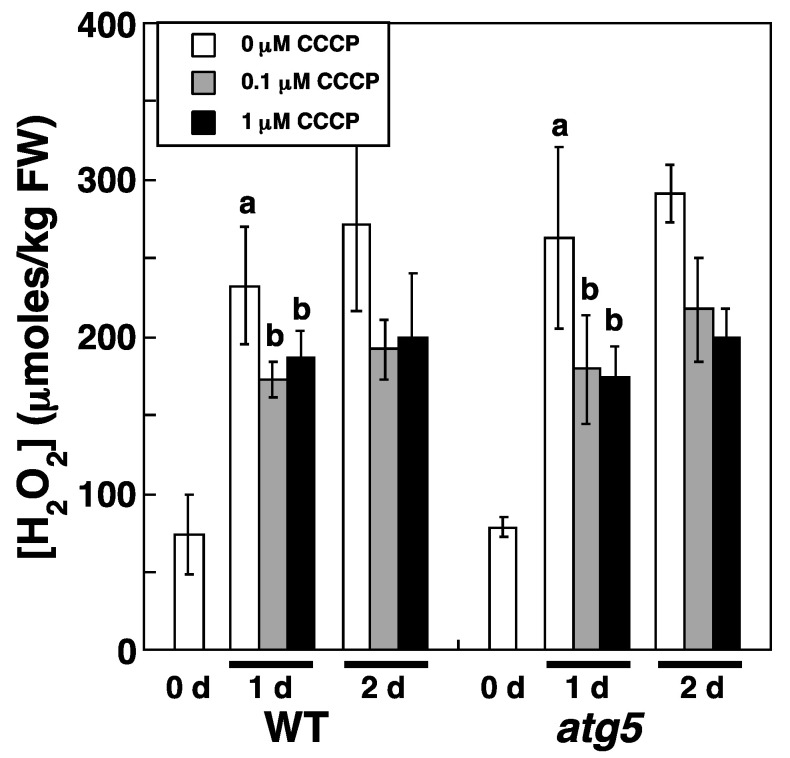
An uncoupler, carbonyl m-chlorophenylhydrazone (CCCP), inhibits the inorganic nitrogen-starvation-induced increase in intracellular H_2_O_2_ levels in WT and *atg5* mutant *Physcomitrium* colonies. WT and *atg5* colonies were transferred onto and cultured on a BCDATG agar medium containing CCCP (0, 0.1, and 1.0 µM) under light conditions. Intracellular H_2_O_2_ levels were measured immediately (0 d), 1 d, and 2 d after the transfer. The data are represented as the means ± SD (n = 3). A two-way ANOVA was performed to analyze the effects of WT or *atg5* and 0, 0.1, or 1 µM CCCP on H_2_O_2_ levels at 1 d and found no significant difference in the interaction between the two strains and the three CCCP concentrations or in H_2_O_2_ levels between WT and *atg5* strains. Therefore, the values obtained in WT and *atg5* strains were pooled to compare the effects of the three CCCP concentrations on H_2_O_2_ levels by one-way ANOVA. Different letters denote significant differences from each other, *p* < 0.005.

We attributed the increase in H_2_O_2_ levels under dark and nitrogen starvation conditions to an increase in ROS production. If ROS production is caused by ETC activity in mitochondria and/or chloroplasts, an uncoupler of the ETC, carbonyl cyanide m-chlorophenylhydrazone (CCCP), should reduce ROS generation, which would in turn reduce H_2_O_2_ levels. Therefore, we investigated whether CCCP affects the increase in cellular H_2_O_2_ levels under inorganic nitrogen starvation conditions. We chose not to investigate the effect of CCCP on H_2_O_2_ levels under dark conditions because H_2_O_2_ levels varied markedly under such conditions (Figure 4). CCCP inhibited the increase in H_2_O_2_ levels that occurs under nitrogen starvation conditions. Specifically, the transfer of WT and *atg5* colonies to an inorganic nitrogen-deprived medium increased H_2_O_2_ levels in both strains from 75–100 to >200 μmoles/kg FW; however, the addition of CCCP (0.1 and 1.0 μM) to the medium reduced this increase in H_2_O_2_ levels in both strains to <200 μmoles/kg FW (Figure 7). This result suggests that ROS production via the ETC in mitochondria and/or chloroplasts is increased in *Physcomitrium* under nitrogen starvation conditions, leading to increased H_2_O_2_ levels.

**Figure 8:**
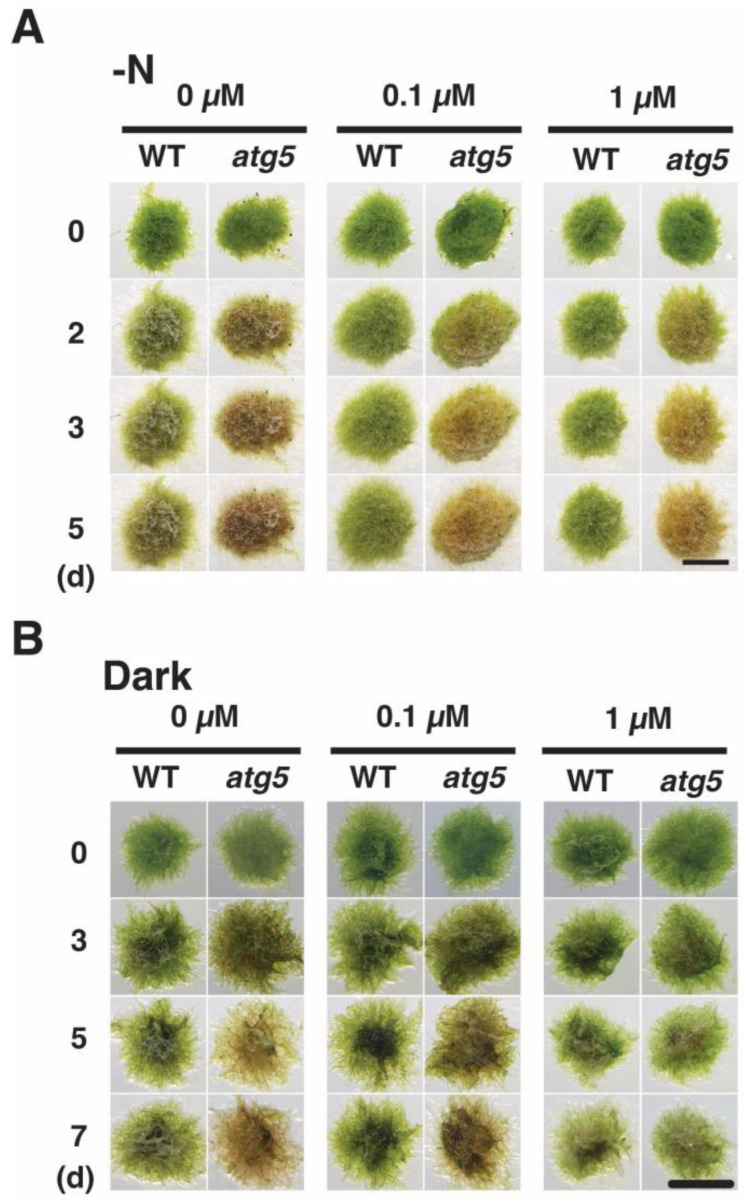
CCCP inhibits inorganic nitrogen-starvation- and dark-induced senescence in WT and *atg5* mutant *Physcomitrium* colonies. (**A**) WT and *atg5* colonies were transferred onto and cultured on an inorganic nitrogen-depleted agar medium containing CCCP (0, 0.1, and 1.0 µM) under light conditions. Individual colonies in each treatment group were successively photographed immediately (0 d), 3, 5, and 7 d after the transfer. (**B**) WT and *atg5* colonies were transferred onto and cultured on a BCDATG agar medium containing CCCP (0, 0.1, and 1.0 µM) under dark conditions. Individual colonies in each treatment group were photographed immediately (0 d), 3, 5, and 7 d after the transfer. Scale bar: 2 mm.

We also investigated the effect of CCCP on yellowing by placing the colonies on inorganic nitrogen-deprived agar medium containing CCCP. In the absence of CCCP, WT colonies yellowed on day 2–5; however, yellowing was inhibited in the presence of CCCP at 0.1 and 1.0 μM (Figure 8A). When CCCP was absent, *atg5* colonies browned on day 2–5, whereas such browning was partially inhibited on the medium containing CCCP at 0.1 and 1.0 μM. CCCP treatment also inhibited darkness-induced yellowing. Specifically, *atg5* colonies became yellow to brown on day 3–5 in the absence of CCCP, but such yellowing was inhibited on the medium containing CCCP at 0.1 and 1.0 μM (Figure 8B). In the absence of CCCP treatment, WT colonies underwent slight yellowing under 7 d of darkness, whereas treatment with CCCP at 0.1 and 1.0 μM maintained the bright green color of the colonies.

These results indicate that CCCP inhibits the progression of darkness- and nitrogen-starvation-induced premature senescence. Thus, we assume that CCCP suppressed the increase in H_2_O_2_ levels and thereby inhibited the progression of senescence.

### 2.8. Elevated H_2_O_2_ Levels Alone Cannot Explain the Difference in the Senescence Responses of WT and atg5 Colonies

**Figure 9:**
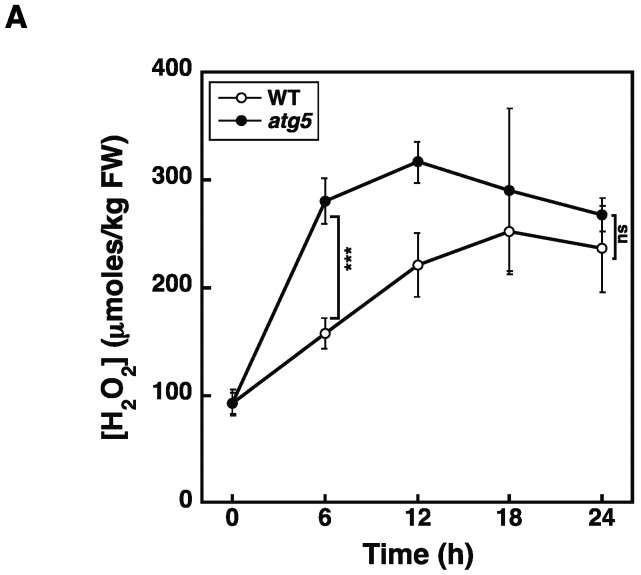

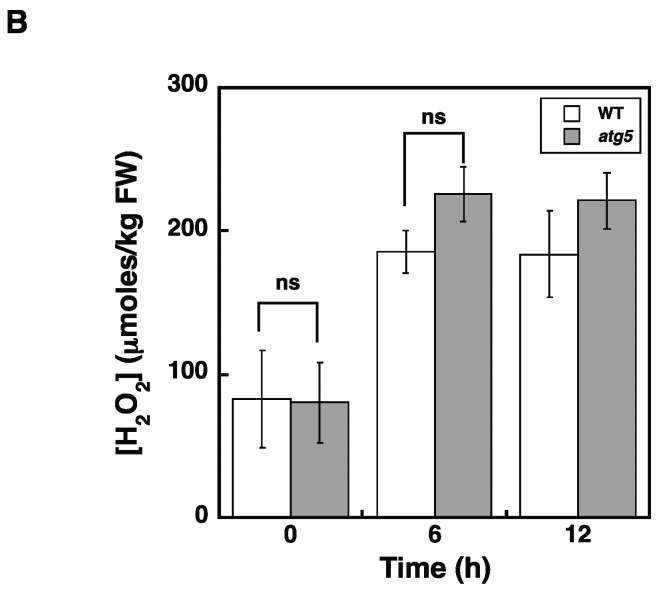
Intracellular H_2_O_2_ levels are increased in *atg5* colonies earlier than those in WT colonies in response to nitrogen starvation. (**A**) WT and *atg5* colonies were transferred onto and cultured on an inorganic nitrogen-depleted agar medium under light conditions. Intracellular H_2_O_2_ levels were measured for 24 h. The data are represented as the means ± SD (n = 3). (**B**) WT and *atg5* colonies were transferred onto and cultured on a BCDATG agar medium containing methyl viologen (0 and 100 µM) under light conditions. Intracellular H_2_O_2_ levels were measured for 12 h. The data are represented as the means ± SD (n = 3, *** *p* < 0.005). ns, not significant.

The results so far strongly suggest that premature senescence induced in the dark and under nitrogen starvation conditions is mediated by elevated levels of H_2_O_2_. However, the onset of both darkness- and nitrogen-starvation-induced senescence occurs earlier and/or the extent of this senescence is higher in the *atg5* mutant relative to the WT strain. In contrast, H_2_O_2_ levels did not differ between *atg5* and WT colonies after 2–3 d following their transfer to the dark or after 1–2 d following their transfer to nitrogen-starvation medium, and these times correspond to the time immediately before yellowing occurred in *atg5* colonies. Therefore, differences in the speed and/or extent of senescence cannot be explained only by differences in H_2_O_2_ levels. However, it remains possible that the timings of the H_2_O_2_ level increase differ between the WT and *atg5* cells. Therefore, we examined a time course of H_2_O_2_ increase after nitrogen starvation treatment. WT and *atg5* colonies were transferred onto nitrogen starvation agar medium, after which they were homogenized at 6 h intervals to measure cellular H_2_O_2_ levels (Figure 9A). We found that the H_2_O_2_ levels in *atg5* cells increased as compared to those in WT cells after 6 h from the transfer to starvation medium and then plateaued. In contrast, the cellular H_2_O_2_ levels in WT cells increased later than in *atg5* cells and reached the same plateau level as in *atg5* cells after 18 h. Therefore, when the supply of inorganic nitrogen from the medium is removed and the nitrogen assimilation pathway is immobilized, the intracellular H_2_O_2_ levels in WT and *atg5* cells increase to reach the same plateau, but *atg5* cells reach this plateau around 12–18 h earlier than it is reached by WT cells. This result shows that differences in the timing and/or degree of progression of nitrogen-starvation-induced senescence between WT and *atg5* can only be partially explained by differences in the timing of the H_2_O_2_ increase. Thus, other factors must be considered when attempting to explain the different senescence responses of WT and *atg5 Physcomitrium*.

We also examined a time course of H_2_O_2_ levels following MV treatment. H_2_O_2_ levels increased in both *Physcomitrium* strains in a similar manner and reached a plateau of around 200 µmoles/kg FW within 6 h (Figure 9B). Considering that senescence symptoms were more severe in *atg5* cells than those in WT cells under nitrogen starvation conditions (Figure 1, **-N**), this result indicates that H_2_O_2_ acts as a trigger rather than as an amplifier, and senescence symptoms, such as yellowing, are somehow attenuated in WT cells.

## 3. Discussion

In this study, we found that MV, an H_2_O_2_ generator, increased cellular H_2_O_2_ levels and reproduced senescence-like symptoms, e.g., yellowing and Rubisco degradation (Figure 2A and Figure 3), in *Physcomitrium* colonies. Furthermore, we showed that cellular H_2_O_2_ levels increased under conditions of darkness and inorganic nitrogen starvation, both of which induced premature senescence. Moreover, we showed that reducing cellular H_2_O_2_ levels using NAC and CCCP treatments decelerates premature senescence. Based on these results, we conclude that H_2_O_2_ is a mediator between senescence-inducing stress, such as darkness and inorganic nitrogen starvation, and the symptoms of senescence, such as yellowing and Rubisco degradation, in *Physcomitrium* cells. We also investigated whether increased H_2_O_2_ levels explain the different senescence responses of the WT strain and *atg5* mutant and concluded that factors other than higher H_2_O_2_ levels should be considered when attempting to explain these differences.

H_2_O_2_ levels increased from 70–100 to 150–200 µmoles/kg FW in *Physcomitrium* cells upon darkness or nitrogen starvation treatment (Figure 4 and Figure 5). Cellular H_2_O_2_ levels are generally thought to be maintained by H_2_O_2_-producing and degrading reactions. Specifically, ROS are produced as byproducts of several metabolic pathways in mitochondria, chloroplasts, and peroxisomes or produced directly by NADPH oxidase, after which they converge with H_2_O_2_, which is decomposed by cellular H_2_O_2_-scavenging enzymes, such as catalase, peroxidase, ascorbate peroxidase, superoxide dismutase and antioxidants including glutathione and ascorbic acid. During natural senescence in *Arabidopsis* and rapeseed, elevated H_2_O_2_ levels are due to decreased levels of catalase and ascorbate peroxidase [5]. Activation of NADPH oxidase also contributes to H_2_O_2_ production in response to osmotic stress in *Arabidopsis* [14]. In our study, increased H_2_O_2_ levels during stress treatment were reduced by CCCP treatment, which is thought to inhibit ROS production via the ETC of mitochondria and chloroplasts, suggesting that the ETCs of these organelles contribute to H_2_O_2_ production under our experimental settings. However, even in the presence of CCCP, H_2_O_2_ levels were higher than those detected before stress treatment (Figure 7), indicating that H_2_O_2_-producing and/or -scavenging reactions other than those that occur via ETCs, including those involving NADPH oxidase, catalase, and ascorbate peroxidase, might also contribute to H_2_O_2_ production.

H_2_O_2_ levels increased and reached the same plateau levels in WT and *atg5* cells under both dark (Figure 4) and inorganic nitrogen starvation conditions (Figure 5), although senescence in *atg5* cells occurred earlier than that in WT cells under both conditions (Figure 1). Therefore, the difference in senescence responses between WT and *atg5* cells, such as the rate and extent of senescence, cannot be explained by differences in H_2_O_2_ plateau levels. The time course of H_2_O_2_ levels after the start of nitrogen starvation treatment varied significantly between WT and *atg5* cells over 18 h (Figure 9A). Specifically, H_2_O_2_ levels were elevated earlier or accelerated in *atg5* cells than in WT cells; however, the difference in the time lag required for H_2_O_2_ levels to reach a plateau was no more than 18 h (Figure 9A), which is not sufficient to explain the time lag in the yellowing response, which is longer than 2–3 d (Figure 1). Similarly, treatment with MV elevated H_2_O_2_ levels in a similar manner to the same plateau levels in WT and *atg5* cells (Figure 3) although the senescence response did differ between the two cell types (Figure 2; Supplemental Figure S1). These results suggest that higher H_2_O_2_ levels act as a trigger of senescence but not as an amplifier of senescence and that the rate and/or extent of senescence is predetermined before the colonies are treated with MV or placed on nutrient-starvation medium. The symptoms of senescence in WT cells are probably alleviated by the constitutive autophagy that occurs in the cells before stress is applied. This notion is supported by the findings in *Physcomitrium* [12], showing that expression of the senescence marker genes *PpSEN1* and *PpSAG12* is higher in *atg5* cells than that in WT cells before dark treatment. Therefore, it is likely that the *atg* mutants were preparing for senescence during culture on nutrient-sufficient medium under light conditions.

It has been shown that accelerated natural senescence in *Arabidopsis atg* mutants is attributable to the higher accumulation of salicylic acid and the consequent activation of salicylic acid signaling; however, even when salicylic acid levels are maintained low by expressing bacterial salicylic acid oxidase in *atg* mutants, dark-induced senescence remains accelerated [7]. In *Physcomitrium* cells cultured on nutrient-sufficient medium under light, salicylic acid levels do not differ significantly between WT and *atg5* cells (personal communication, Dr. Seo). Thus, we still need to determine the factors that are responsible for the difference of premature senescence responses between WT and *atg5* cells.

## 4. Materials and Methods

### 4.1. Biological Materials

The WT and *atg5* strains of *Physcomitrium patens* were cultured on BCDATG agar medium overlaid with cellophane [12,15]. Once per week, colonies consisting of protonemal cells were collected and transferred onto fresh BCDATG agar medium. Seven-day-old colonies maintained at 25 °C on the medium under continuous illumination from fluorescent lights (5–7 w/m^2^) were used in this study. For the nitrogen starvation treatment, we used an inorganic nitrogen-depleted BCDATG medium, in which KNO_3_ was removed and ammonium tartrate was replaced with potassium tartrate in the BCDATG medium.

### 4.2. H_2_O_2_ Measurement

H_2_O_2_ content was measured using the ferrous ammonium sulfate/xylenol orange (FOX) method with a few modifications [16]. Approximately 0.05 g fresh weight of colonies was homogenized with 1 mL 25 mM H_2_SO_4_. The homogenate was centrifuged at 15,000× *g* for 5 min. Subsequently, 100 µL of the resulting supernatant was mixed with 1 mL of FOX solution consisting of 150 µM ammonium ferrous sulfate (Nacalai Tesque, Inc., Kyoto, Japan), 150 µM xylenol orange (398187, Sigma-Aldrich, Burlington, MA, USA) and 100 mM sorbitol (Nacalai Tesque, Inc., Kyoto, Japan) in 25 mM H_2_SO_4_. The mixture was incubated in the dark for 20 min, after which A_560_ was measured. Commercially available H_2_O_2_ (084-07441, Fujifilm Wako Pure Chemical Corporation, Osaka, Japan) was used as a standard.

### 4.3. SDS-PAGE

Around 0.05 g fresh weight of colonies was homogenized with a solution containing 0.1 M HEPES-Na (pH 7.5), 1 mM EDTA, 10 µM antipain, 1 mM 4-(2-Aminoethyl) benzenesulfonyl fluoride hydrochloride (AEBSF; Nacalai Tesque, Inc., Kyoto, Japan), and 14 mM 2-mercaptoethanol in a mortar and pestle on ice. The homogenates were centrifuged at 15,000× *g* and 4 °C for 10 min. The resulting supernatant was collected and mixed with the same volume of 2-fold-concentrated SDS sample buffer (Cosmo Bio Corporation, Tokyo, Japan), and the mixture was boiled at 100 °C for 2 min. The proteins were then loaded into each lane on the basis of FW and separated on SDS-polyacrylamine gels (10%, Ready Gel model E-T10L e-PAGEL, Atto Corporation, Tokyo, Japan), after which they were stained using Coomassie brilliant blue R-250 (Merck, Rahway, NJ, USA). Relative intensities of the Rubisco band were quantitated using Image J (im-agej.nih.gov/ij/download/). The highest value of the band was set as 100%.

### 4.4. Statistical Analysis

Statistical analysis was performed using KaleidaGraph (Synergy, Stroudsburg, PA, USA). Two groups were compared using Student’s *t*-test, whereas multiple group comparisons were performed using ANOVA with Tukey’s test. Differences were considered statistically significant at * *p* < 0.05, ** *p* < 0.01 or *** *p* < 0.005.

## 5. Conclusions

During premature senescence under dark or nitrogen starvation conditions in *Physcomitrium* cells, increased intracellular H_2_O_2_ mediates between stress perception and senescence responses. However, increased H_2_O_2_ levels do not explain the earlier senescence phenotype in autophagy-deficient mutants.

## Data Availability

All datasets generated for this study are included in the article/Supplementary Materials and further inquiries can be directed to the corresponding author.

## Supplementary Materials

The following supporting information can be downloaded at: https://www.mdpi.com/article/10.3390/plants11172280/s1, Supplemental Figure S1. Senescence-like symptoms in *atg3*, *atg7*, and *ATG5* (in which the *Phycomitrium ATG5* gene was introduced into the *atg5*-3 mutant) mutant *Physcomitrium* colonies were induced by methyl viologen (MV) treatment under light conditions. Colonies of *atg3*, *atg7*, and *ATG5* mutants were transferred onto and cultured on a BCDATG agar medium containing MV (0, 100, and 500 µM) under light conditions. Individual colonies in each treatment group were photographed successively immediately (0 d), 3, 5, and 7 d after the transfer. Scale bar: 2 mm; Supplemental Figure S2. Senescence-like symptoms in WT and *atg5* mutant *Physcomitrium* colonies cultured in liquid culture medium were induced by methyl viologen (MV) under light and dark conditions. WT and *atg5* colonies were transferred onto and cultured in a BCDATG liquid medium containing MV (0 and 100 µM) under light (left) and dark (right) conditions. Individual colonies in each treatment group were photographed immediately (0 d), 3, 5, and 7 d after the transfer. Scale bar: 2 mm.
