# Supplementary material for: Hydrogen Peroxide Mediates Premature Senescence Caused by Darkness and Inorganic Nitrogen Starvation in Physcomitrium patens
Source: Plants (Basel). 2022 Aug 31;11(17):2280. doi: 10.3390/plants11172280 (PMC9460043; doi:10.3390/plants11172280)
Supplement: Supplementary file 1 [file plants-11-02280-s001.zip › plants-1842010-supplementary.pdf]

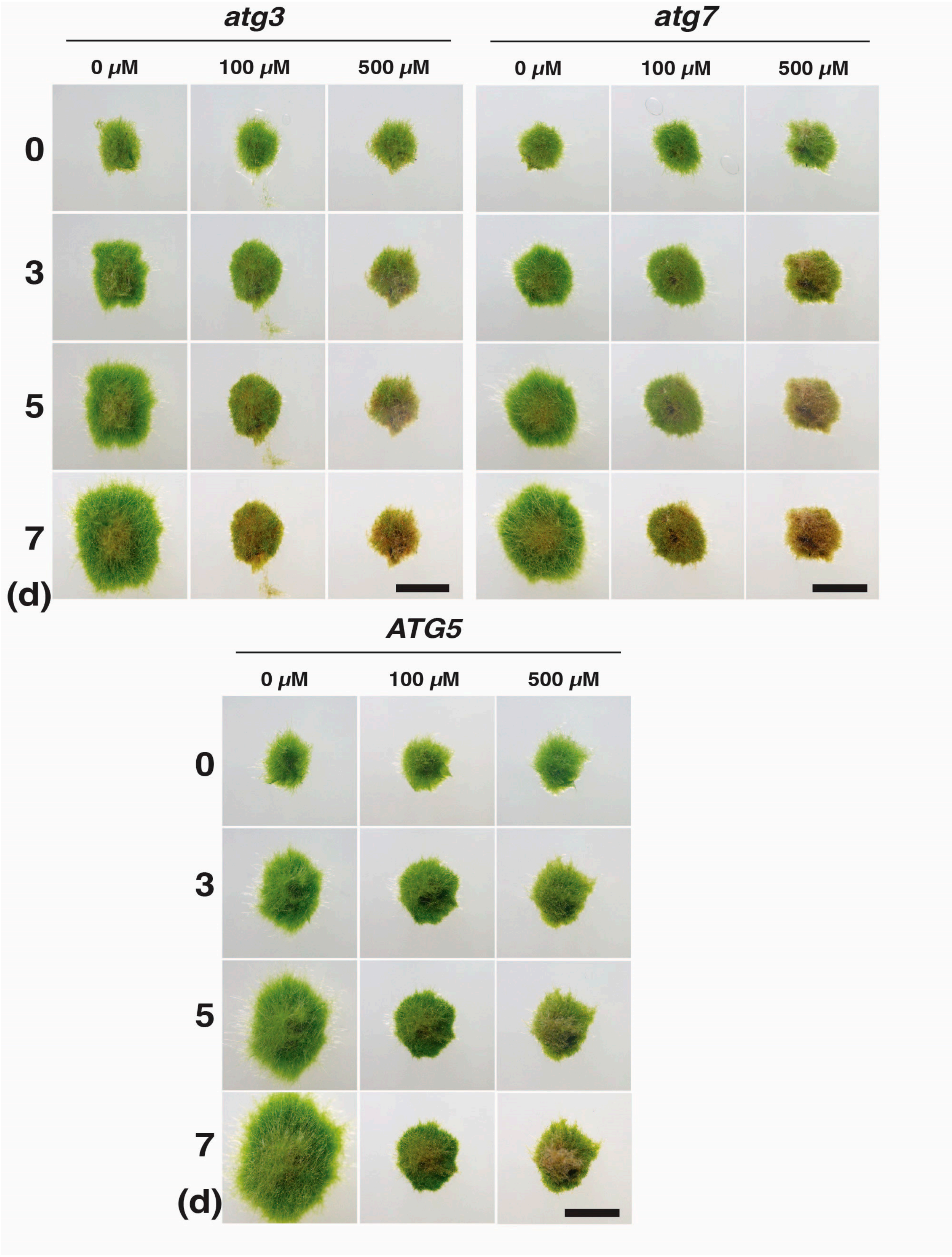

Figure S1. Senescence-like Symptoms in *atg3,atg7,* and *ATG5* mutant *Physcomitrella* colonies were induced by methyl viologen (MV) treatment under light conditions.

Colonies of *atg3,atg7,* and *ATG5* mutants were transferred noto and clutured on a BCDATG agar medium containing MV (0, 100, and 500 uM) under light conditions. Individual colonies in each treatment group were photographed successively immediately (0 d), 3, 5, and 7 d after transfer. Scale bar: 2 mm.

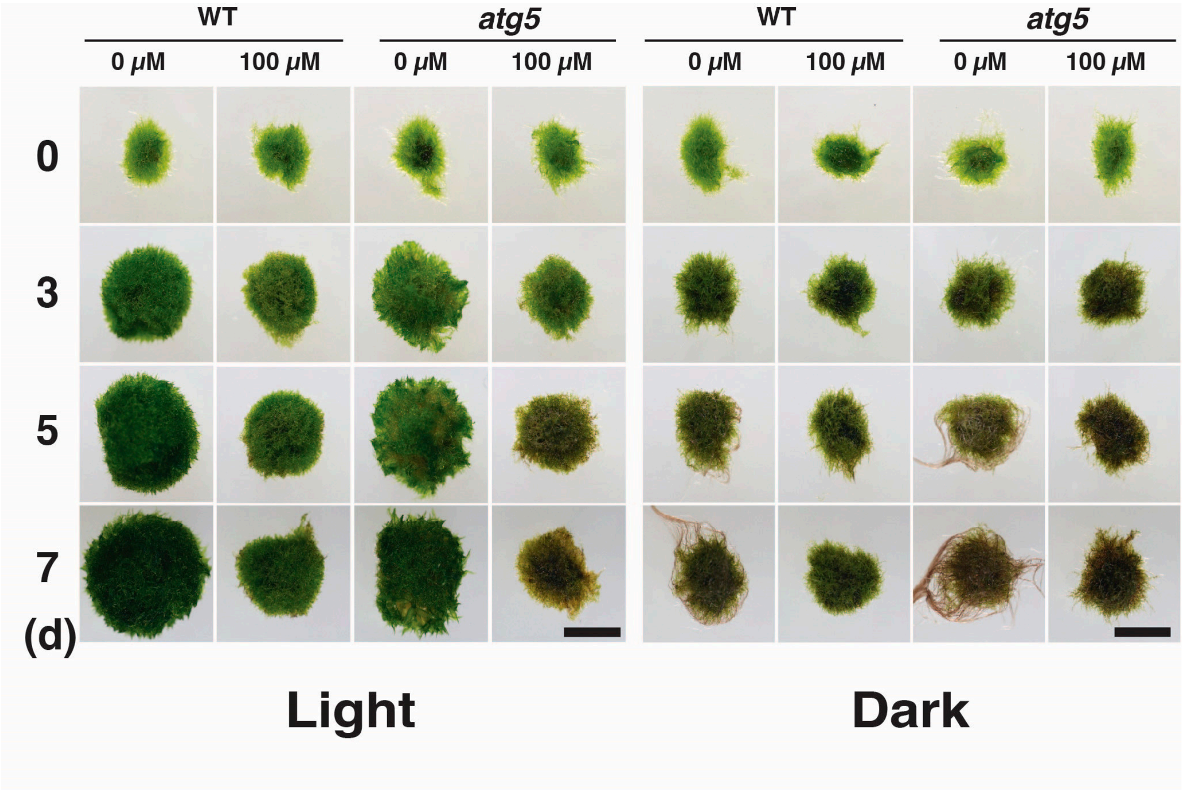

Figure S2. Senescence-like Symptoms in WT and *atg5* mutant *Physcomitrella* colonies clutured in liquid culture medium were induced by methyl viologen (MV) under light and dark conditions.

WT and *atg5* colonies were transferred noto and clutured on a BCDATG liquid medium containing MV (0 and 100 uM) under light (left) and dark (right) conditions. Individual colonies in each treatment group were photographed successively immediately (0 d), 3, 5, and 7 d after transfer. Scale bar: 2 mm.
